# In Memory of Dr. James Taylor

**Published:** 1881-07

**Authors:** H. A. Smith, J. Taft, James Leslie


### EDITORIAL.

### IN MEMORY OF DR. JAMES TAYLOR.

At a meeting of the members of the dental profession, held in the lecture room of the Ohio Dental College, Tuesday evening, June 14th, 1881, the following action was taken:

Dr. W. S. How was chosen Chairman, and Dr. A. G. Rose, Secretary.

A committee of three, consisting of Drs. H. A. Smith, J. Taft, and James Leslie was appointed to draft a communication expressing the sympathy of the profession of the city, in reference to the death of Dr. Taylor. After consideration, the following was presented and adopted, viz:

"Death is a common event among men. Some pass away after much suffering, longing for its release of pain; but the sudden death of our dear friend and teacher, Professor James Taylor, awakes within us feelings of profound sorrow. But a day before his death, and in his apparent usual health, some of us conversed with him about the affairs of the Ohio College of Dental Surgery, of which it may be said, he was pre-eminently its founder, and one of its professors, ever since his indomitable energy and professional spirit brought it into being, thirty-six years ago; and during all that time his interest never abated. In its infancy he nursed it with all the tenderness of a mother for her child, and he lived long enough to have a pride in its present strength. Strange, indeed, that so far away from all the seats of learning, he founded the first dental college in the world. Thousands of dollars he gave for which he received no returns, and years of faithful and laborious services for which he received no pay. He gathered others around him, inspiring them with a portion of his spirit; and during the day of its early struggles continually made sacrifices for the uplifting of the profession he had adopted, and diffusing among men educated practitioners of the dental profession. He was one of the few living practitioners who are remembered as the fathers of our profession; and the Alumni scattered all over this country, and in every nation of the globe, will respond with the feeling of genuine sorrow when they hear that their revered instructor is dead. We remember his urbanity—his gentle, manly deportment, his kind words, his ability, and patience as a teacher; and though the years were passing. time left her mark so gently on him, that his compeers failed to realize he had reached the ripe age of seventy-two.

Notwithstanding a large, private practice he could not ignore, last winter he met his classes with much of their own youthful vigor, and lectured with all the ability of his more youthful years, showing himself familiar with the latest discoveries and improvements pertaining to our profession. He gathered diligently and gave freely of all he had acquired as a teacher, culled or established by a ripe experience; but if there is one act more than another, in addition to all his labor for the education of others, that prominently marks the character of this noble man, it was that feeling that prompted him to give to every graduate, with reverence, a copy of the Bible. The diploma he conferred was the evidence of their professional standing; but this last gift indicated a study that remineded them of something above the curriculum of college studies.

His daily life was a fine example of its teachings. We thank God for the beautiful life of our friend as illustrated during so many years in science, in the church, in the State, and the private walks of men.

In his death we have some conception of the deep sorrow that dwells in the heart of the bereaved widow, and relatives; and we tender to her and them our deep sympathy in this sudden bereavement.

H. A. Smith,
J. Taft,
James Leslie.

After the adoption of the report, many of the members gave expression to feelings of sadness and sorrow. The words spoken also gave indication of great respect and honor for the deceased brother.

